# The value of secondary neoadjuvant chemotherapy in platinum-sensitive recurrent ovarian cancer: a case-control study post GOG-0213 trial

**DOI:** 10.1186/s13048-020-00673-0

**Published:** 2020-06-16

**Authors:** Hongyuan Gu, Rui Zhou, Jing Ni, Xia Xu, Xianzhong Cheng, Yan Li, Xiaoxiang Chen

**Affiliations:** 1 Department of Gynecologic Oncology, The Affiliated Cancer Hospital of Nanjing Medical University, Jiangsu Cancer Hospital, Jiangsu Institute of Cancer Research, Nanjing, 210009 Jiangsu P.R. China; 2 Nanjing Gaochun People’s Hospital, Nanjing, 211300 Jiangsu P.R. China; 3 Department of Chemotherapy, The Affiliated Cancer Hospital of Nanjing Medical University, Jiangsu Cancer Hospital, Jiangsu Institute of Cancer Research, Nanjing, 210009 Jiangsu P.R. China; 4 The Medical College of Yangzhou University, Yangzhou, Jiangsu P.R. China

**Keywords:** Epithelial ovarian cancer, Platinum sensitive recurrent ovarian cancer, Secondary cytoreductive surgery, Secondary neo-adjuvant chemotherapy, Time to progression, Platinum free interval

## Abstract

**Background:**

The prognostic value and optimal resection outcome related factors of the secondary cytoreduction surgery (SCR) in Platinum-sensitive recurrent ovarian cancer (PSOC) patients were still in doubt. The present retrospective study aims to determine the relationship between the objective response of secondary neo-adjuvant chemotherapy (SNAC) and the resection outcome of SCR.

**Methods:**

Data were reviewed from 142 type II PSOCs who underwent SCR in Jiangsu Institute of Cancer Research between 1996 and 2016. Among them, 55 cases received preliminary Platinum based SNAC before SCR. Logistic regression analysis was used to explore optimal SCR related factors. Cox proportional hazards model and log-rank test were used to assess the associations between the survival durations and covariates.

**Results:**

Optimal initial CRS (*p* = 0.02), disappearance of ascites after SNAC (*p* = 0.04) recurrent status (*p* = 0.02) and longer Platinum-free interval (*p* = 0.01) were the independent indicators of optimal SCR. Optimal SCR was associated with time to progression (TTP) but not overall survival (OS) (*p* = 0.04 and *p* = 0.41). The TTP and OS of PSOCs underwent SNAC were similar to those patients underwent SCR (*p* = 0.71, and *p* = 0.77, respectively) directly.

**Conclusions:**

SNAC might be another choice for PSOCs were not suitable for directly SCR. Optimal SCR had survival benefit in PSOCs whenever underwent SNAC or not.

## Background

The standard primary treatment of Epithelial ovarian cancer (EOC) includes cytoreductive surgery and Platinum based chemotherapy. Though more than 50% of EOC patients results in a complete clinical response (CCR) through initial therapy, about 75% EOC patients develop recurrent disease within 2 years [1]. The mean 5-year survival rate following the recurrence is less than 10% [2]**.** Therefore, the management of recurrent ovarian cancer (ROC) is of primary importance.

There was no consensus on the standard treatment of women with ROC. DESKTOP III trial suggested a survival benefit of SCR, while Gynecologic Oncology Group Protocol (GOG) 213 stated that SCR improves neither PFS nor OS [3–6]. Different from DESKTOP III, the patients with resectable disease (to no macroscopic residual disease) recruited for SCR were investigator-determined. The randomization process is somewhat flawed. Presently, salvage chemotherapy with or without antiangiogenic agents and secondary cytoreductive surgery (SCR) were deemed to be the main approaches for Platinum-sensitive recurrent ovarian cancer (PSOC). Compared to chemotherapy only, optimal SCR may be a chance to improve objective response and another longer interval. Complete cytoreductive surgery has been thought to be the cornerstone of PSOC patients’ management [7–12]. However, the factors affect the outcomes of SCR has not been fully revealed. Exploring the potential beneficial subpopulation and selection criteria of optimal SCR is indispensable. Presently, the adequate selection of women with PSOC for surgery is crucial due to the primary goal of SCR aiming at achieving complete gross resection [13–15]. Several score systems such as AGO, Tian and MSK [16–18] was used to enroll PSOCs for this potentially morbidity risk procedure. Our previous study revealed that biochemical relapse guided asymptomatic PSOC optimized the SCR [19].

Practically, a SCR procedure could be considered in patients with recurrent ovarian cancer who recur more than 6–12 months since completion of initial chemotherapy, have an isolated focus (or limited foci) of disease amenable to complete resection, and do not have ascites [20]. Other than SCR directly, there were patients who underwent secondary neo-adjuvant chemotherapy (SNAC) and followed secondary debulking surgery. When patients with a high perioperative risk profile or a low likelihood of achieving optimal surgery outcome identified by multidisciplinary team (MDT) [21], chemotherapy was the best choice left. After several cycles of Platinum based chemotherapy and the appearance of objective response, the possibility of SCR will be discussed again by MDT. In this situation, SNAC was expected to reduce the patient’s ascites or even tumor burden and improve therapeutic effect by allowing further surgery. Presently, there was no consensus on whether the response to SNAC is associated with the outcomes of SCR and the prognosis in PSOCs.

Primary objective of the present study was to evaluate the impact of the SCR on time to progression (TTP) and overall survival (OS) in women with PSOC undergoing SNAC and following SCR compared to those who receiving SCR directly. Other prognostic factors influenced the survival were also investigated. Furthermore, characteristics affecting the outcome of SCR were also analyzed to reveal those who potential benefit with the opportunity for this procedure.

## Methods

### Study population

This retrospective study was approved by the ethics committee of the Jiangsu Institute of Cancer Research (JICR). Informed consent was obtained from all involved participants. One hundred forty-two type II PSOCs who underwent SCR at the Department of Gynecology Oncology in JICR were identified between January 1, 1996 and December 31, 2016. Among them, 55 cases underwent preliminary SNAC.

Those who did not undergo the standard first line treatment and achieved objective response were excluded. Multidisciplinary team (MDT) including two gynecologic oncologists, two pathologists, one radiologist and one medical oncologist was set to identify patients with a high perioperative risk profile or a low likelihood of achieving optimal cytoreduction should receive SNAC. The number of SNAC cycles was administered based on the MDT’s decision. The select criteria of SCR was mainly according to a modified AGO [16] model whenever patients underwent SNAC or not. Briefly, the recruited standard includes, the disease-free interval of more than 6–12 months since completion of primary therapy, have an isolated focus (or limited foci) of disease amenable to complete resection, and do not have ascites.

SRS as a selective procedure was performed in patients with good performance status (ECOG 0–1) and intended purpose of tumor resection. The routine follow-up protocol was described previously. The Response Evaluation Criteria in Solid Tumors (RECIST) criterion was used to assess therapy response and tumor progression [22, 23]. The clinicopathological data such as, the histological type and grade of the tumor, stage of the disease, volume of ascites, management of the disease, and the survival information were collected. Pathological materials of all recruited cases were independently assessed by two pathologists, namely, Xu and Hou.

### Definition of clinical response and surveillance

In brief, CCR was defined according to the following criteria: absence of tumor-associated clinical symptoms and residual tumor on the physical examination, absence of EOC-negative imaging study results on CT, MRI, or PET; or no increase of post-treatment serum CA-125 concentration below the upper limit of the normal range (ULN = 35 U/mL). Clinical recurrent was identified as the reappearance of any lesion, or the development of a new lesion considered to be malignant through imaging studies or clinical examination [22]**.** Platinum-sensitive recurrent is defined as the recurrence of active disease in a patient who has achieved a documented response to initial platinum-based treatment and has been off therapy for at least 6 months from the completion of primary therapy. SCR is defined as an additional debulking procedure performed at some time remote other than during the completion of primary treatment. Type of resection was defined as follows: R0-no remnant tumoral tissue, R1-tumoral tissue between 0 and 1 cm, and R2-tumoral tissue > 2 cm. The criterion of optimal cytoreduction was the threshold of residual tumor ≤1 cm or macroscopic free. Progression-free survival (PFS) was defined as the duration wherein the patient’s condition does not worsen after initial therapy. The overall survival (OS) was the length of the time from the disease diagnosis to death or last follow-up. Conventionally, recurrent ovarian cancer has been classified on the basis of platinum-free interval (PFI), which is calculated from the last cycle of platinum-containing treatment to the time of disease progression. Though the last Gynecologic Cancer Intergroup consensus conference proposed to replace PFI with the term therapy-free interval (TFI) to better define different trial populations, present study applied the 2011 Gynecologic Cancer Intergroup categorization as most clinical trials in recurrent disease [24, 25]. The length of time from the date of radiological defined relapse until the disease starts to get worse or spread to other parts of the body was called TTP in present study.

### Statistical analysis

Cox proportional hazards model was used to assess the survival rerated clinical characteristics. Step-wise regression was conducted to build the multivariate models. Logistic regression analysis was used to explore optimal SCR related factors. The *p* values < 0.05 was considered statistically significant. All analyses were conducted using the SPSS statistical software program (version 20.0; SSPS Inc., Chicago, IL).

## Results

### Patient characteristics

The clinicopathological characteristics of all 142 type II PSOCs recruited were given in Table 1. The most common subtype was of high-grade serous carcinoma (73.2%). Median follow-up time was 47.5 months (interquartile range, 22.8 months to 70.4 months). The commonly reported complications or adverse effect of SCR were pain (3 cases), gastrointestinal dysfunction (10 cases), mass effect (9 cases) and/or others (12 cases). In all 142 type II PSOCs, 55 (38.7%) cases underwent SNAC and subsequent cytoreduction procedure.

**Table 1:** Patient characteristics of the study population

Characteristic	Percentage (%)/Median (range)	
Age (years)	62.7	(32–85)
**Baseline CA-125 level (U/mL)**	725	(14–26,190)
**Nadir CA-125 level (U/mL)**	10	(6–35)
**CA-125 level at relapse (U/mL)**	124	(32–5528)
**Histology**		
High grade Serous	104	(73.2)
High grade Endometrioid	26	(18.3)
Undifferentiated	9	(6.3)
Malignant mixed müllerian tumor	3	(2.1)
**Surgical residual of initial CRS**		
< 1 cm	88	(62.0)
1–2 cm	5	(3.5)
> 2 cm	28	(19.7)
Unknown	21	(14.8)
**FIGO stage**		
I	10	(7.0)
II	11	(7.7)
III	95	(63.6)
IV	20	(14.1)
Unknown	6	(4.2)
**NAC**	92	(63.4)
**SNAC**	55	(38.7)
**BRCA mutations**	5	(22.7)
**Avastin® at recurrence**	44	(31.0)

FIGO the International Federation of Gynecology and Obstetrics, *NAC* Neo-adjuvant chemotherapy, *SNAC* Secondary Neo-adjuvant chemotherapy

### Optimal SCR associated factors in PSOCs

The optimal resection rates were similar in patients underwent SNAC (20.0%) and SRC directly (21.8%). There was also no difference in the serious perioperative complications rate (10.4% vs. 8.5%, respectively) between these two groups, and those in mortality rate (1.8% vs. 0.0%, respectively). To explore the potential factors related to optimal SCR, we performed multivariate logistic regression analysis in PSOCs, we found that optimal initial CRS (*p* = 0.02), longer Platinum-free interval (*p* = 0.01), recurrent status (*p* = 0.02) and response to SNAC (*p* = 0.04) were the independent indicators for optimal resection (as seen in Table 2).

**Table 2:** Logistic regression of optimal SCR associated factors in type II PSOCs

Variable	Univariate		Multivariate	
	Exp (B)	Sig	Exp (B)	Sig
Age	1.02	0.04	1.00	0.35
Ascites	2.43	0.00	1.25	0.10
Optimal initial CRS	5.67	0.00	4.10	0.02
PFI	3.12	0.01	2.68	0.01
Recurrent status	1.96	0.00	1.84	0.02
SNAC	1.80	0.00	1.63	0.04
Stage	4.45	0.04	2.46	0.61
CA-125 level at relapse	1.00	0.00	1.01	0.45

*SCR* secondary cytoreduction, *CRS* cytoreduction surgery, *PSOCs* Platinum-sensitive recurrent ovarian cancer, *PFI* Platinum free interval, *NAC* Neo-adjuvant chemotherapy, *SNAC* Secondary Neo-adjuvant chemotherapy

Recurrent status including symptom or biomarker indicated relapse

### Survival related factors in PSOCs

Outcome of initial and secondary cytoreductions, response to SNAC and PFI were associate with TTP and OS in all recruited PSOCs by univariate Cox proportional hazards mode. Multivariate analysis revealed that optimal SCR, response to SNAC could independently predict TTP and OS in PSOCs (Table 3).

**Table 3:** Survival-related characteristics in type II PSOCs underwent SCR

Variable	Univariate		Multivariate	
	TTP (OR 95%CI)	OS (OR 95%CI)	TTP (OR 95%CI)	OS (OR 95%CI)
FIGO stage				
I-II	1.00(reference)	1.00(reference)	1.00(reference)	1.00(reference)
III-IV	3.55 (1.89–10.05)	4.18 (2.42–11.27)	2.13 (0.93–4.28)	3.24 (0.83–4.28)
Ascites	1.85 (1.35–2.54)	1.99 (1.38–2.33)	1.54 (1.25–2.06)	1.85 (0.83–2.25)
Optimal initial CRS	6.33 (2.26–16.08)	6.20 (3.53–15.06)	5.08 (2.45–15.25)	6.12 (3.44–14.67)
Optimal SCR	4.81 (1.98–13.21)	5.95 (3.45–17.26)	4.68 (1.85–13.48)	6.28 (4.20–16.60)
NAC	1.12 (0.96–1.49)	1.25 (0.77–2.27)	1.19 (0.94–1.47)	1.25 (0.82–2.21)
SNAC	1.10 (0.73–1.28)	1.29 (0.80–2.02)	1.05 (0.87–1.35)	1.21 (0.88–1.97)
PFI	1.08 (1.00–1.15)	1.12 (1.08–1.25)	1.08 (1.00–1.17)	1.12 (1.07–1.26)
Nadir CA-125	1.01 (1.00–1.03)	1.02 (1.00–1.05)	1.02 (1.00–1.02)	1.03 (1.00–1.05)

*TTP* time to progression, *OS* overall survival

**Fig. 1:**
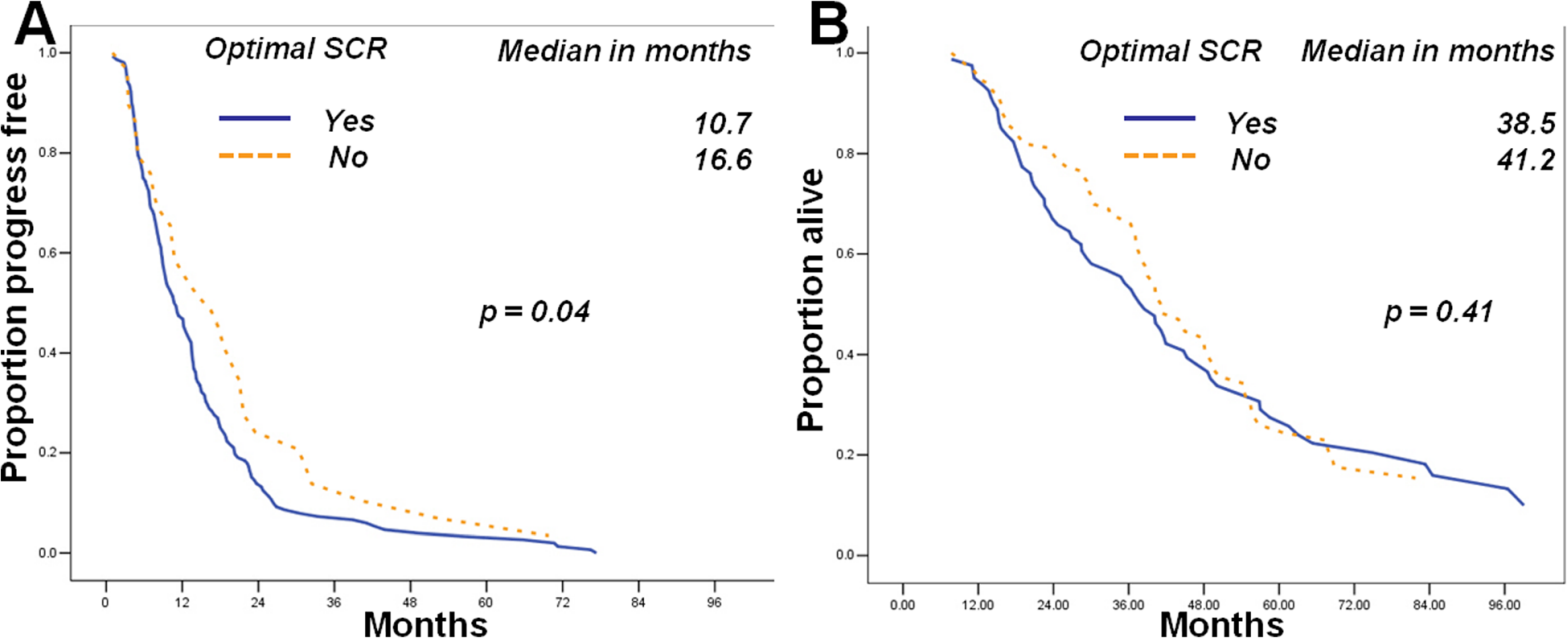
Patients who underwent optimal SCR had longer TTP durations (**a**) than those who did not undergo optimal surgery, but there was no difference on OS (**b**)

**Fig. 2:**
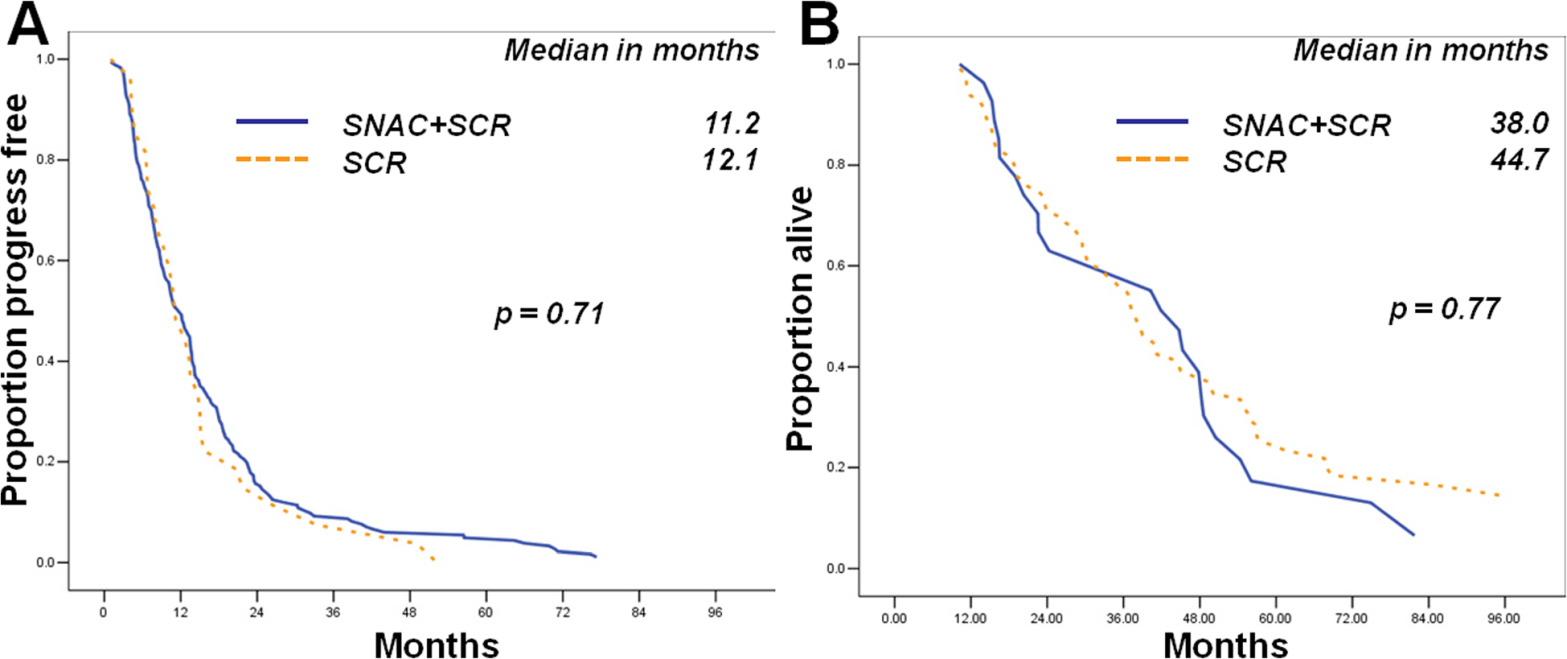
Patients who underwent SNAC and SCR had similar TTP (**a**) and OS durations (**b**) to those who underwent SCR directly

The TTP durations of recurrent ovarian cancer patients who underwent optimal SCR were comparatively longer than those of suboptimal group (*p* = 0.04, Fig. 1a), but not OS (*p* = 0.41 Fig. 1b). In PSOCs underwent SCR, the TTP and OS durations of recurrent ovarian cancer patients underwent SNAC were similar to those of cases who underwent SCR directly (*p* = 0.71 and *p* = 0.77 respectively; Fig. 2a and b).

## Discussion

Presently, combination Platinum based chemotherapy with or without antiangiogenic agents is deemed the main approach in therapy for PSOC [26]**.** The following PARP inhibitors maintenance therapy following complete or partial remission cases was deemed to be the latest development in this subgroup [27–31]. However, SCR may also have the irreplaceable role in the management of some recurrent ovarian cancer under special circumstances [32]**.** In most cases, SCR approach was considered in PSOCs with no ascites and isolated focus (or limited foci) of disease amenable to complete resection. Practically, there were some patients underwent chemotherapy (SNAC) and achieved objective response, and thus to be candidate cases for SCR due to the opinion by MDT. While respecting inclusion criteria for SCR, we firstly demonstrated a significant improvement in TTP in SNAC subgroup. Furthermore, in all SCR groups, zero macroscopic residual disease after SCR were identified as favorable prognostic factors for both PFS and OS in a multivariate analysis.

Though being explored by several non-RCT studies, the utility criterion and the prognostic role of SCR were still indeterminate [11, 33–39]. DESKTOP III study suggested a survival benefit of SCR, especially outstanding in patients with complete resection with a prolonged PFS of 5.6 months while combined with adjuvant chemotherapy (*P* < 0.001) [3]. On the contrary, Gynecologic Oncology Group Protocol 213 stated that secondary CRS improves neither PFS nor OS in patients underwent initial optimal surgery [4]. The OS was even comparatively lower in patients underwent SCR than that of counterparts though without statistical difference. To finally clarify this controversy, a multicenter randomized double-blind controlled trial on this issue from Netherlands named SOCcer was conducted. It’s regrettable that the trial was premature stop for the low number of patients up to recruited standards [40]. Therefore, the influence of SCR on PFS and OS was still unclear. In our previous studies, tumor grade, ascites, nadir serum CA 125 level, tumor residual after SCR and PFS were independent prognostic factors for TTP and OS in patients underwent this procedure [19, 41]. Berek et al. proved that tumor size was associated with survival while Park et al. denied this relationship in PSOCs [8, 42]. Other factors including performance status, preoperative and post-operative chemotherapy, histologic type, elevated CA 125 level and number of tumors at recurrence has been reported to be prognostic factors [7, 33, 39].

Besides the survival benefit of SCR in selected PSOCs, who will be appropriate for optimal SCR is another extremely important concern on this topic. Residual tumor, ascites and progression-free interval durations were proved to indicate to be associated with surgical outcomes in most studies [8, 11, 43, 44]. On the contrary, other studies found these factors had no impact of on the surgical residual of SCR [7, 10, 45, 46]. Zang et al. stated that PSOCs with solitary lesions, no ascites, achieved initial optimal surgical outcomes will be benefit more easily for SCR in a large population more than one thousand cases [33, 34, 47]. Our previous series studies proved that CA-125 indicated asymptomatic recurrent cases will benefit for optimal secondary CRS [19, 41].

Besides residual burden, morbidity and mortality rates during perioperative period were also important. In general, SCR was considered to be a safe procedure in the management of selected PSOCs [8, 48, 49]. Postoperative morbidity rates were reported to be ranged from 5 to 35% [8, 36, 39]. In present study, there was only one SCR related death.

There are limitations to the present study. First of all, selection biases inherent to all retrospective researches cannot be avoided. The response to SNAC may have reflected certain selected factors that may influence surgery residual and prognosis, though we eliminate the influence by inclusion criteria. Secondly, given the20 years follow up durations, new agents such as bevacizumab (antiangiogenic agent) and PARP inhibitors maintenance therapy. Thirdly, the inclusion standard was set by different MDTs and absence of unified recruited standard for SCR and limited sample size for a single institutional study. Fourthly, populations underwent SCR was comparatively with better ECOG performance status, and a high likelihood of enduring SNAC and postoperative chemotherapy. Present retrospective research cannot be easily translated to all PSOCs until further proved by prospectively randomized controlled trials.

## Conclusion

In summary, present study found that SNAC followed by SCR has survival benefit to selected patients. The survival durations of patients underwent SANC and secondary cytoreduction were no less than those of patients underwent SCR directly. Some selected PSOCs may get a chance of SCR after chemotherapy.

## Data Availability

We would not share the data and material used in this manuscript, because we need them for further research.

## Abbreviations

CCR: Complete clinical response
OS: Overall survival
PFS: Progression-free survival
TTP: Time to progression
OR: Odds ratio
CI: Confidence interval
EOC: Epithelial ovarian cancer
CRS: Cytoreductive surgery
ULN: Upper limit of normal
RECIST: Response Evaluation Criteria in Solid Tumors

## References

[1] Siegel RL, Miller KD, Jemal A (2019). Cancer statistics, 2019. CA Cancer J Clin.

[2] Cannistra SA (2004). Cancer of the ovary. N Engl J Med.

[3] Du Bois A, Vergote I, Ferron G, et al. Randomized controlled phase III study evaluating the impact of secondary cytoreductive surgery in recurrent ovarian cancer: AGO DESKTOP III/ENGOT ov20. Am Soc Clin Oncol. 2017.

[4] Coleman RL, Enserro D, Spirtos N, et al. A phase III randomized controlled trial of secondary surgical cytoreduction (SSC) followed by platinum-based combination chemotherapy (PBC), with or without bevacizumab (B) in platinum-sensitive, recurrent ovarian cancer (PSOC): a NRG oncology/gynecologic oncology group (GOG) study. Am Soc Clin Oncol. 2018.

[5] Coleman RL, Spirtos NM, Enserro D (2019). Secondary surgical Cytoreduction for recurrent ovarian cancer. N Engl J Med.

[6] Coleman RL, Brady MF, Herzog TJ (2017). Bevacizumab and paclitaxel-carboplatin chemotherapy and secondary cytoreduction in recurrent, platinum-sensitive ovarian cancer (NRG oncology/gynecologic oncology group study GOG-0213): a multicentre, open-label, randomised, phase 3 trial. Lancet Oncol.

[7] Benedetti Panici P, De Vivo A, Bellati F (2007). Secondary cytoreductive surgery in patients with platinum-sensitive recurrent ovarian cancer. Ann Surg Oncol.

[8] Park JY, Eom JM, Kim DY (2010). Secondary cytoreductive surgery in the management of platinum-sensitive recurrent epithelial ovarian cancer. J Surg Oncol.

[9] Landoni F, Pellegrino A, Cormio G (1998). Platin-based chemotherapy and salvage surgery in recurrent ovarian cancer following negative second-look laparotomy. Acta Obstet Gynecol Scand.

[10] Boran N, Hizli D, Yilmaz S (2012). Secondary cytoreductive surgery outcomes of selected patients with paclitaxel/platinum sensitive recurrent epithelial ovarian cancer. J Surg Oncol.

[11] Chi DS, McCaughty K, Diaz JP (2006). Guidelines and selection criteria for secondary cytoreductive surgery in patients with recurrent, platinum-sensitive epithelial ovarian carcinoma. Cancer.

[12] Bristow RE, Puri I, Chi DS (2009). Cytoreductive surgery for recurrent ovarian cancer: a meta-analysis. Gynecol Oncol.

[13] Harter P, Heitz F, du Bois A (2012). Surgery for relapsed ovarian cancer: when should it be offered?. Curr Oncol Rep.

[14] Sehouli J, Richter R, Braicu EI (2010). Role of secondary cytoreductive surgery in ovarian cancer relapse: who will benefit? A systematic analysis of 240 consecutive patients. J Surg Oncol.

[15] Suh DH, Kim HS, Chang SJ (2016). Surgical management of recurrent ovarian cancer. Gynecol Oncol.

[16] Harter P, du Bois A, Hahmann M (2006). Surgery in recurrent ovarian cancer: the Arbeitsgemeinschaft Gynaekologische Onkologie (AGO) DESKTOP OVAR trial. Ann Surg Oncol.

[17] Harter P, Hahmann M, Lueck HJ (2009). Surgery for recurrent ovarian cancer: role of peritoneal carcinomatosis: exploratory analysis of the DESKTOP I trial about risk factors, surgical implications, and prognostic value of peritoneal carcinomatosis. Ann Surg Oncol.

[18] Tian WJ, Chi DS, Sehouli J (2012). A risk model for secondary cytoreductive surgery in recurrent ovarian cancer: an evidence-based proposal for patient selection. Ann Surg Oncol.

[19] Wang F, Ye Y, Xu X (2013). CA-125-indicated asymptomatic relapse confers survival benefit to ovarian cancer patients who underwent secondary cytoreduction surgery. J Ovarian Res.

[20] O'Malley DM (2019). New therapies for ovarian cancer. J Natl Compr Cancer Netw.

[21] Burton E, Chase D, Yamamoto M (2011). Surgical management of recurrent ovarian cancer: the advantage of collaborative surgical management and a multidisciplinary approach. Gynecol Oncol.

[22] Therasse P, Arbuck SG, Eisenhauer EA (2000). New guidelines to evaluate the response to treatment in solid tumors. European Organization for Research and Treatment of cancer, National Cancer Institute of the United States, National Cancer Institute of Canada. J Natl Cancer Inst.

[23] Eisenhauer EA, Therasse P, Bogaerts J (2009). New response evaluation criteria in solid tumours: revised RECIST guideline (version 1.1). Eur J Cancer.

[24] Friedlander M, Trimble E, Tinker A (2011). Clinical trials in recurrent ovarian cancer. Int J Gynecol Cancer.

[25] Wilson M, Pujade-Lauraine E, Aoki D (2016). Fifth ovarian Cancer consensus conference of the gynecologic cancer InterGroup: recurrent disease. Ann Oncol.

[26] Aghajanian C, Goff B, Nycum LR (2015). Final overall survival and safety analysis of OCEANS, a phase 3 trial of chemotherapy with or without bevacizumab in patients with platinum-sensitive recurrent ovarian cancer. Gynecol Oncol.

[27] Pujade-Lauraine E, Ledermann JA, Selle F (2017). Olaparib tablets as maintenance therapy in patients with platinum-sensitive, relapsed ovarian cancer and a BRCA1/2 mutation (SOLO2/ENGOT-Ov21): a double-blind, randomised, placebo-controlled, phase 3 trial. Lancet Oncol.

[28] Friedlander M, Gebski V, Gibbs E (2018). Health-related quality of life and patient-centred outcomes with olaparib maintenance after chemotherapy in patients with platinum-sensitive, relapsed ovarian cancer and a BRCA1/2 mutation (SOLO2/ENGOT Ov-21): a placebo-controlled, phase 3 randomised trial. Lancet Oncol.

[29] Oza AM, Matulonis UA, Malander S (2018). Quality of life in patients with recurrent ovarian cancer treated with niraparib versus placebo (ENGOT-OV16/NOVA): results from a double-blind, phase 3, randomised controlled trial. Lancet Oncol.

[30] Del Campo JM, Matulonis UA, Malander S (2019). Niraparib maintenance therapy in patients with recurrent ovarian cancer after a partial response to the last platinum-based chemotherapy in the ENGOT-OV16/NOVA trial. J Clin Oncol.

[31] Matulonis UA, Walder L, Nottrup TJ (2019). Niraparib maintenance treatment improves time without symptoms or toxicity (TWiST) versus routine surveillance in recurrent ovarian cancer: a TWiST analysis of the ENGOT-OV16/NOVA trial. J Clin Oncol.

[32] Al Rawahi T, Lopes AD, Bristow RE (2013). Surgical cytoreduction for recurrent epithelial ovarian cancer. Cochrane Database Syst Rev.

[33] Zang RY, Li ZT, Tang J (2004). Secondary cytoreductive surgery for patients with relapsed epithelial ovarian carcinoma: who benefits?. Cancer.

[34] Zang RY, Zhang ZY, Li ZT (2000). Effect of cytoreductive surgery on survival of patients with recurrent epithelial ovarian cancer. J Surg Oncol.

[35] Cheng X, Jiang R, Li ZT (2009). The role of secondary cytoreductive surgery for recurrent mucinous epithelial ovarian cancer (mEOC). Eur J Surg Oncol.

[36] Gungor M, Ortac F, Arvas M (2005). The role of secondary cytoreductive surgery for recurrent ovarian cancer. Gynecol Oncol.

[37] Onda T, Yoshikawa H, Yasugi T (2005). Secondary cytoreductive surgery for recurrent epithelial ovarian carcinoma: proposal for patients selection. Br J Cancer.

[38] Scarabelli C, Gallo A, Carbone A (2001). Secondary cytoreductive surgery for patients with recurrent epithelial ovarian carcinoma. Gynecol Oncol.

[39] Eisenkop SM, Friedman RL, Spirtos NM (2000). The role of secondary cytoreductive surgery in the treatment of patients with recurrent epithelial ovarian carcinoma. Cancer.

[40] Kruitwagen R, Zusterzeel P, Van TG (2017). Correspondence: premature stop of the SOCceR trial, a multicenter randomized controlled trial on secondary Cytoreductive surgery: Netherlands trial register number: NTR3337. Int J Gynecol Cancer.

[41] Xu X, Chen X, Dai Z (2013). Secondary cytoreduction surgery improves prognosis in platinum-sensitive recurrent ovarian cancer. J Exp Clin Cancer Res.

[42] Berek JS, Hacker NF, Lagasse LD (1983). Survival of patients following secondary cytoreductive surgery in ovarian cancer. Obstet Gynecol.

[43] Salani R, Santillan A, Zahurak ML (2007). Secondary cytoreductive surgery for localized, recurrent epithelial ovarian cancer: analysis of prognostic factors and survival outcome. Cancer.

[44] Tebes SJ, Sayer RA, Palmer JM (2007). Cytoreductive surgery for patients with recurrent epithelial ovarian carcinoma. Gynecol Oncol.

[45] Munkarah AR, Coleman RL (2004). Critical evaluation of secondary cytoreduction in recurrent ovarian cancer. Gynecol Oncol.

[46] Munkarah A, Levenback C, Wolf JK (2001). Secondary cytoreductive surgery for localized intra-abdominal recurrences in epithelial ovarian cancer. Gynecol Oncol.

[47] Zang RY, Harter P, Chi DS (2011). Predictors of survival in patients with recurrent ovarian cancer undergoing secondary cytoreductive surgery based on the pooled analysis of an international collaborative cohort. Br J Cancer.

[48] Kim K, Ryu SY (2009). Prognostic factors of secondary cytoreductive surgery for patients with recurrent epithelial ovarian cancer. J Gynecol Oncol.

[49] Bae J, Lim MC, Choi JH (2009). Prognostic factors of secondary cytoreductive surgery for patients with recurrent epithelial ovarian cancer. J Gynecol Oncol.

